# Deep learning reconstruction with single-energy metal artifact reduction in pelvic computed tomography for patients with metal hip prostheses

**DOI:** 10.1007/s11604-023-01402-5

**Published:** 2023-03-02

**Authors:** Reina Hosoi, Koichiro Yasaka, Masumi Mizuki, Haruomi Yamaguchi, Rintaro Miyo, Akiyoshi Hamada, Osamu Abe

**Affiliations:** 1 Department of Radiology, The University of Tokyo Hospital, 7-3-1 Hongo, Bunkyo-ku, Tokyo, 113-8655 Japan; 2 Department of Radiology, Juntendo University Urayasu Hospital, 2-1-1 Tomioka, Urayasu, Chiba 279-0021 Japan; 3 Department of Radiology, Nerimahikarigaoka Hospital, 2-11-1 Hikarigaoka, Nerima-ku, Tokyo, 179-0072 Japan; 4 Department of Radiology, The Institute of Medical Science Hospital, The University of Tokyo, 4-6-1 Shirokanedai, Minato-ku, Tokyo, 108-8639 Japan; 5 Department of Radiology, International University of Health and Welfare Narita Hospital, 852 Hatakeda Narita, Chiba, 286-8520 Japan

**Keywords:** Metal artifact reduction, Metal hip prosthesis, Pelvis, Computed tomography

## Abstract

**Purpose:**

The aim of this study was to assess the impact of the deep learning reconstruction (DLR) with single-energy metal artifact reduction (SEMAR) (DLR-S) technique in pelvic helical computed tomography (CT) images for patients with metal hip prostheses and compare it with DLR and hybrid iterative reconstruction (IR) with SEMAR (IR-S).

**Materials and methods:**

This retrospective study included 26 patients (mean age 68.6 ± 16.6 years, with 9 males and 17 females) with metal hip prostheses who underwent a CT examination including the pelvis. Axial pelvic CT images were reconstructed using DLR-S, DLR, and IR-S. In one-by-one qualitative analyses, two radiologists evaluated the degree of metal artifacts, noise, and pelvic structure depiction. In side-by-side qualitative analyses (DLR-S *vs.* IR-S), the two radiologists evaluated metal artifacts and overall quality. By placing regions of interest on the bladder and psoas muscle, the standard deviations of their CT attenuation were recorded, and the artifact index was calculated based on them. Results were compared between DLR-S *vs.* DLR and DLR *vs.* IR-S using the Wilcoxon signed-rank test.

**Results:**

In one-by-one qualitative analyses, metal artifacts and structure depiction in DLR-S were significantly better than those in DLR; however, between DLR-S and IR-S, significant differences were noted only for reader 1. Image noise in DLR-S was rated as significantly reduced compared with that in IR-S by both readers. In side-by-side analyses, both readers rated that the DLR-S images are significantly better than IR-S images regarding overall image quality and metal artifacts. The median (interquartile range) of the artifact index for DLR-S was 10.1 (4.4–16.0) and was significantly better than those for DLR (23.1, 6.5–36.1) and IR-S (11.4, 7.8–17.9).

**Conclusion:**

DLR-S provided better pelvic CT images in patients with metal hip prostheses than IR-S and DLR.

## Introduction

In recent years, the number of people undergoing hip replacement surgery has increased [1, 2]. The indications for hip replacement surgery mainly cover a wide range of diseases such as hip joint osteoarthritis, femoral neck fracture, femoral head osteonecrosis, and rheumatoid arthritis [1]. Degenerative changes in the hip joint are the primary cause, and the number of patients undergoing hip arthroplasty is still expected to increase as the global population ages. Elderly people have more comorbidities and are more likely to undergo diagnostic imaging such as computed tomography (CT) and magnetic resonance imaging (MRI). The CT is mostly performed for screening purposes, because CT has the advantages of superior accessibility and the ability to be performed in patients with contraindications to MRI imaging (e.g., those with MRI incompatible devices, claustrophobia, etc.). However, image quality deterioration due to metal artifacts is a problem in patients after hip arthroplasty.

Various attempts have been made in the past to reduce metal artifacts. For example, increasing the tube voltage or tube current can increase photon penetration or reduce photon starvation, respectively, and these result in improved CT image quality. However, at the same time, it has the disadvantage of increasing radiation exposure [3]. Since the 2010s, single-energy metal artifact reduction (SEMAR) has been introduced in clinical practice, resulting in images with reduced metal artifacts [4, 5]. This technique could be used in combination with the hybrid iterative reconstruction (IR) algorithm, and IR with SEMAR (IR-S) is known to be effective in reducing metal artifacts in patients with metal hip prostheses compared with IR [6, 7].

Recently, deep learning applications have been gaining wide attention in the radiology field [8]. Recent studies have shown that deep learning allows not only imaging diagnosis [9] but also image processing [10]. Deep learning reconstruction (DLR) is one of such algorithms. DLR is known to reduce noise in CT images when compared to IR [11–13]. Therefore, it is expected that DLR will be widely applied to daily clinical practice, replacing IR. DLR can be used in combination with the SEMAR algorithm. However, there have been no reports that assessed the usefulness of DLR with SEMAR (DLR-S) images in patients with hip prostheses.

The purpose of this study was to assess the DLR-S algorithm’s usefulness in pelvic CT for patients with metal hip prostheses by comparing it with the DLR and IR-S algorithms.

## Materials and methods

This retrospective study was approved by our Research Ethics Committee, and the requirement for obtaining written informed consent was waived.

### Patients

We searched the picture archiving and communication system (PACS) for all the consecutive patients with metal in the hip joint who underwent CT including the pelvic region using a 320-row multidetector CT scanner (Aquilion ONE; Canon Medical Systems, Tochigi, Japan). We identified 26 consecutive patients [mean age 68.6 ± 16.6 years, 9 men (mean age 62.7 ± 23.6 years) and 17 women (mean age 67.8 ± 10.5 years)] between November 2021 and February 2022, and they were included in the analysis. The hip metal location was bilateral (*n* = 3), right-sided (*n* = 9), and left-sided (*n* = 14). The CT imaging indications were the following: malignancy evaluation (*n* = 14), metal hip prosthesis evaluation (*n* = 5), contralateral hip joint preoperative evaluation (*n *= 2), liver transplant donor (*n* = 1), enlarged lymph nodes examination (*n* = 1), screening for malignancy (*n* = 1), suspected infection (*n* = 1), and deep vein thrombosis (*n* = 1).

### CT imaging

All the patients underwent CT examination using Aquilion ONE (Canon Medical Systems, Otawara, Japan). The CT scanning parameters were the following: tube voltage, 120 kVp; tube current, automatic tube current modulation was used with the standard deviation (SD) set at 13.0; helical pitch, 0.813; and gantry rotation time, 0.5 s. There were 10 unenhanced and 16 enhanced CT examinations. The concentration and volume of the contrast agent were selected according to body weight. In patients with renal dysfunction, the contrast agent dose was reduced appropriately. The time interval between the start of injection and scan was 90 s.

The mean CT dose index and dose-length product (with scan range) were 8.04 ± 1.69 mGy and 861.8 ± 206.9 mGy cm (neck to femur, 5 patients), 44.8 ± 59.6 mGy and 1094.5 ± 698.2 mGy cm (chest to femur, 11 patients), 77.2 ± 0 mGy and 808.1 ± 0 mGy cm (abdomen to femur, 1 patient), 7.8 ± 1.8 mGy and 475.2 ± 131.6 mGy cm (lower extremity, 5 patients), and 12.3 ± 6.1 mGy and 932.9 ± 319.9 mGy cm (chest to lower extremity, 4 patients), respectively.

### CT image reconstruction

From the helical scan data, axial images of the pelvis were reconstructed using DLR-S (Advanced Intelligent Clear-IQ Engine with body sharp standard [Canon Medical Systems] and SEMAR [Canon Medical Systems]), DLR (Advanced Intelligent Clear-IQ Engine with body sharp standard), and IR-S (Adaptive Iterative Dose Reduction enhanced standard with FC03 kernel [Canon Medical Systems]). The following image reconstruction parameters were the same in all the reconstruction algorithms: slice thickness, 3.0 mm; slice interval, 3.0 mm; field of view, 35–40 cm (adjusted to body size); and Z-axis range, from the iliac crest to the ischial tuberosity.

The CT images were anonymized and exported from the PACS in digital imaging and communications in medicine format.

### Quantitative image analyses

**Fig. 1:**
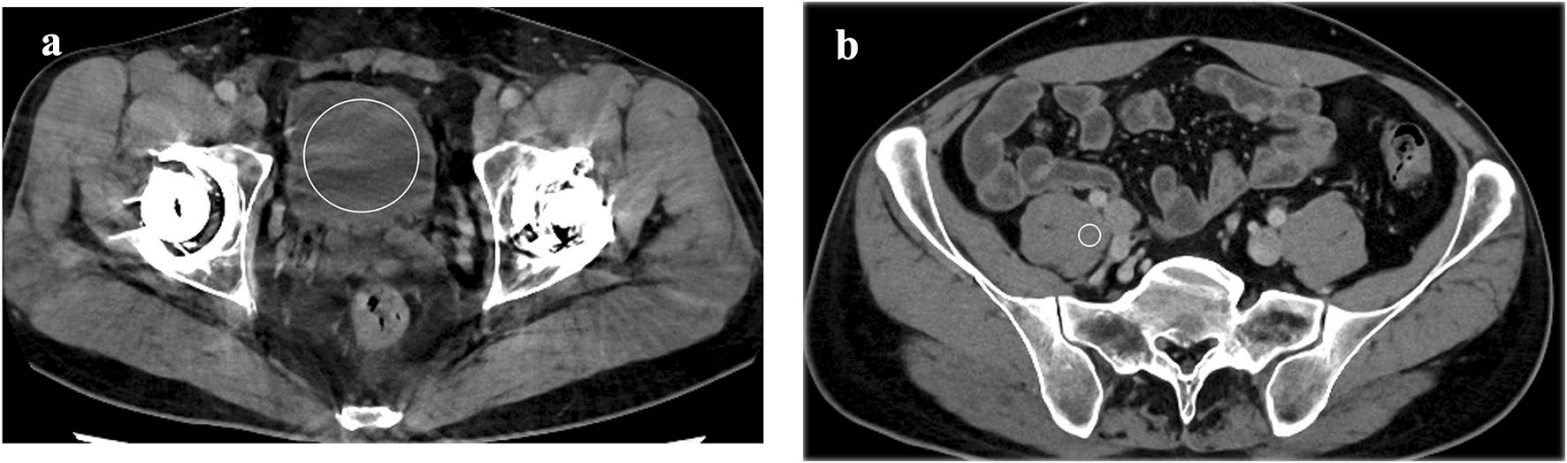
Axial contrast-enhanced CT images reconstructed with DLR-S (**a**, **b**) of a 41-year-old male. For quantitative evaluation, circular or oval regions of interest (ROI) were placed on the bladder at the slice where noise is prominent (**a**) and the psoas muscle at the slice where metals are absent (b). In the bladder, the ROI was placed as large as possible so as not to include the bladder wall (**a**). In the psoas muscle, the ROI diameter was kept to be approximately 10 mm (**b**)

A radiologist (with imaging experience of 3 years) performed the quantitative image analysis using Image J (http://imagej.nih.gov/ij). Circular or oval regions of interest (ROIs) were placed on the bladder at the slice where noise was prominent and the psoas muscle at the slice where metals were absent (Fig. 1). In the bladder, the ROI size was set as large as possible so as not to include the bladder wall. In the psoas muscle, the ROI diameter was kept to be approximately 10 mm. The copy and paste function of the ROI was used to ensure that the ROI location and the size were the same across all the three reconstruction algorithms. The SDs of the CT attenuation in the bladder (SD_B_) and muscle (SD_M_) were recorded. Then, the artifact index was calculated using the following formula [14]:

$${\text{Artifact index}}{\mkern 1mu} = {\mkern 1mu} \left( {{\text{SD}}_{{\text{B}}}^{2} {\mkern 1mu} - {\mkern 1mu} {\text{SD}}_{{\text{M}}}^{2} } \right)^{{1/2}} {\text{(when SDB > SDM) or 0 (when SDB < SDM)}}.$$

### Qualitative image analyses (one-by-one)

In qualitative image analyses, two other radiologists (readers 1 and 2, with 7-year and 2-year imaging experience, respectively) were involved. All image sets were randomly ordered and evaluated one-by-one using ImageJ. They were blinded to the information of patient clinical data and reconstruction algorithms. The two radiologists independently evaluated the image sets in terms of the following:

- Structure depiction (bladder, ureters, rectum, rectovesical/rectouterine pouch, pelvic sidewalls, and nearby bones) on a 5-point scale (5 = clear depiction, 4 = slightly blurred, 3 = moderately blurred, 2 = noticeably blurred, and 1 = unrecognizable)
- Metal artifacts on a 5-point scale (5 = no artifact, 4 = minimal artifacts, 3 = moderate artifacts, 2 = severe artifacts in a small area, and 1 = severe artifacts in a large area)
- Subjective image noise on a 5-point scale (5 = almost no noise, 4 = less than standard noise, 3 = standard noise, 2 = more than standard noise, and 1 = severe noise) (by focusing on bladder and/or subcutaneous adipose tissue in image slice where metal is absent)
- Overall image quality on a 5-point scale (5 = excellent, 4 = better than standard, 3 = standard, 2 = worse than standard, and 1 = poor)

### Qualitative image analyses (side-by-side)

Next, to detect subtle difference in the degree of artifacts and overall quality between DLR-S and IR-S, these two images were compared in a side-by-side way. Because IR-S was known to be superior to IR [6, 7], we assumed that the superiority of DLR-S over DLR could be detected in the one-by-one qualitative analyses. Therefore, we omitted the comparison of DLR-S vs. DLR in this analysis. The places of these images (left or right) were changed randomly for each patient, and the two readers were blinded to the respective reconstruction methods. Both the artifacts and overall image quality were rated on a 5-point scale (− 2 = left image is clearly better, − 1 = left image is rather better, 0 = left and right is the same, 1 = right image is rather better, and 2 = right image is clearly better).

### Statistical analysis

Statistical analyses were performed using EZR version 1.55 (https://www.jichi.ac.jp/saitama-sct/SaitamaHP.files/statmed.html) [15], which is a graphical user interface of R version 4.2.0 (https://www.r-project.org/) (R foundation for Statistical Computing, Vienna, Austria).

The results for the quantitative and one-by-one qualitative analyses were compared using the Wilcoxon signed-rank test between DLR-S *vs.* IR-S and DLR-S *vs.* DLR with a priori comparison strategy. Because of multiple comparisons, the Bonferroni correction was applied. Values of *p* less than 0.05 were considered statistically significant differences.

For the one-by-one qualitative analyses, interobserver agreements between the two readers were evaluated using Cohen’s weighted kappa analysis (quadratic weight was used). For 10 patients who were randomly selected out of 26 patients, the two readers re-evaluated the images, and intraobserver agreements were also evaluated. Poor, fair, moderate, good, and excellent agreement corresponded to the ranges of kappa values of 0.00–0.20, 0.21–0.40, 0.41–0.60, 0.61–0.80, and 0.81–1.00, respectively.

In the side-by-side qualitative image analysis, scores were adjusted so that DLR-S would have a positive value if it is better than IR-S and vice versa. The 95% confidence interval for DLR-S was calculated. When the lower limit of the 95% confidence interval exceeded 0, it indicated the significant superiority of DLR-S over IR-S.

## Results

### Quantitative image analyses

Results for quantitative image analyses are summarized in Table 1. The SD_M_ median value, which is an image noise indicator, in DLR-S (7.9) was significantly lower than that in IR-S (10.5) (*p* < 0.001). There was no significant difference in SD_M_ between DLR-S and DLR (7.7) (*p* = 1.000). The SD_B_ median value in DLR-S, DLR, and IR-S were 13.1, 24.2, and 16.7, respectively. There were significant differences between DLR-S *vs.* DLR and DLR-S *vs.* IR-S (p < 0.001 for both).

**Table 1:** Results for quantitative image analyses

	Median values (with quartile ranges)			Comparisons (*p*-values)	
	DLR-S	DLR	IR-S	DLR-S *vs.* DLR	DLR-S *vs.* IR-S
SD_B_	13.1 (8.8–18.1)	24.2 (10.7–36.8)	16.7 (12.2–21.0)	< 0.001*	< 0.001*
SD_M_	7.9 (6.7–8.9)	7.7 (6.8–8.9)	10.5 (8.5–11.9)	1.000	< 0.001*
Artifact index	10.1 (4.4–16.0)	23.1 (6.5–36.1)	11.4 (7.8–17.9)	< 0.001*	< 0.001*

*DLR* deep learning reconstruction, *DLR-S* deep learning reconstruction with single-energy metal artifact reduction, *IR-S* hybrid iterative reconstruction with single-energy metal artifact reduction, *SD*_*B*_ standard deviation of the CT attenuation in the bladder, *SD*_*M*_ standard deviation of the CT attenuation in the muscle

The Wilcoxon signed-rank test was used to perform comparisons

*Statistically significant difference (*p* < 0.05)

The median value of the artifact index in DLR-S (10.1) was significantly lower than those in DLR (23.1) and IR-S (11.4) (*p* < 0.001 for both).

### Qualitative image analyses (one-by-one)

**Fig. 2:**
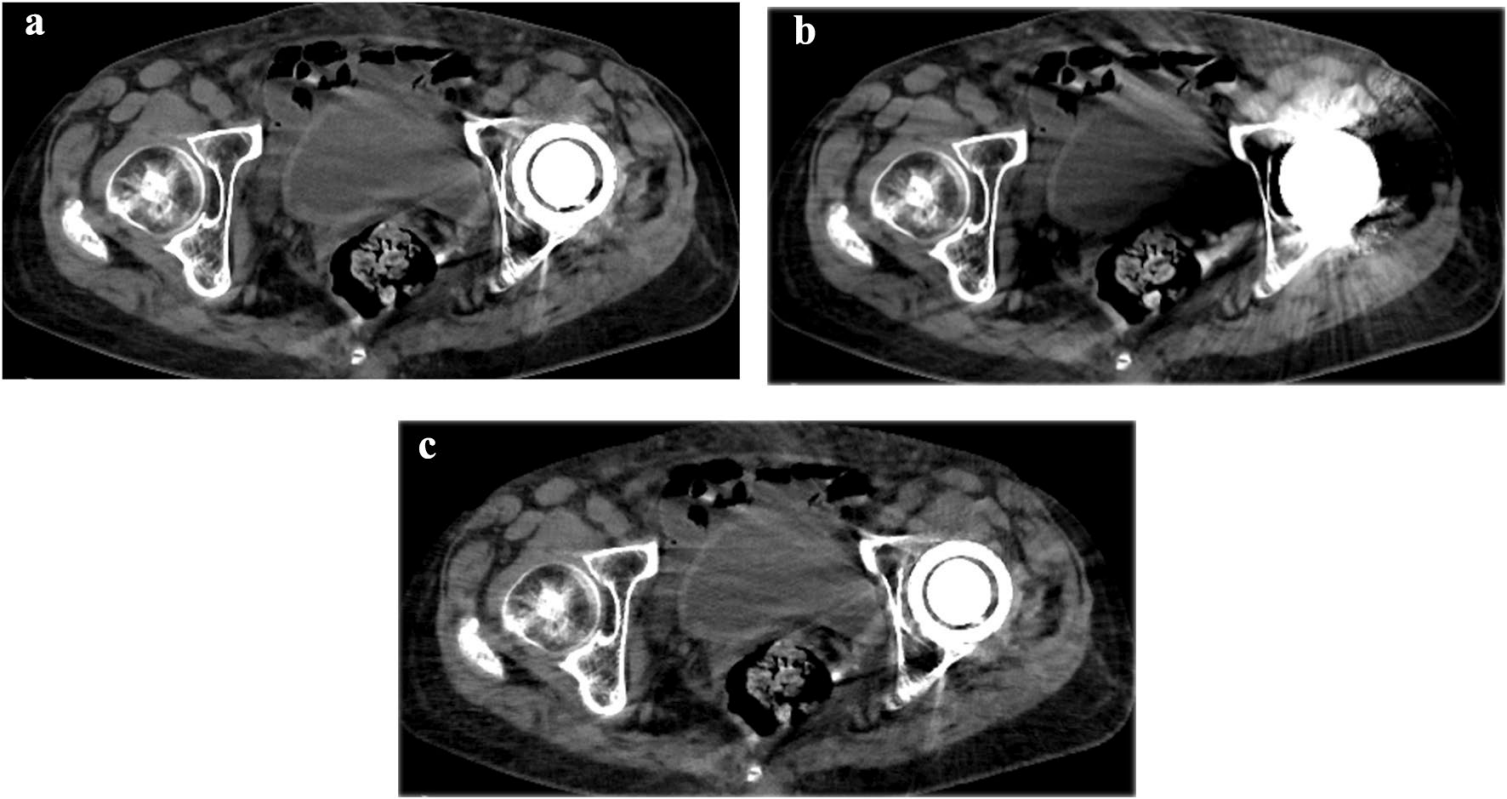
Axial unenhanced CT images reconstructed with DLR-S (**a**), DLR (**b**), and IR-S (**c**) of an 84-year-old woman. The degree of metal artifacts (DLR-S/DLR/IR-S) was rated 4 (minimal artifacts)/1 (severe artifacts in a large area)/2 (severe artifacts in a small area) by reader 1. It was rated 3 (moderate artifacts)/2 (severe artifacts in a small area)/3 (moderate artifacts) by reader 2. Bladder delineation (DLR-S/DLR/IR-S) was rated 3 (moderately blurred)/1 (unrecognizable)/2 (notably blurred) by reader 1. Reader 2 rated it as 2 (noticeably blurred)/2 (noticeably blurred)/3 (moderately blurred). No lesions were noted in the artifact slice in this case

**Fig. 3:**
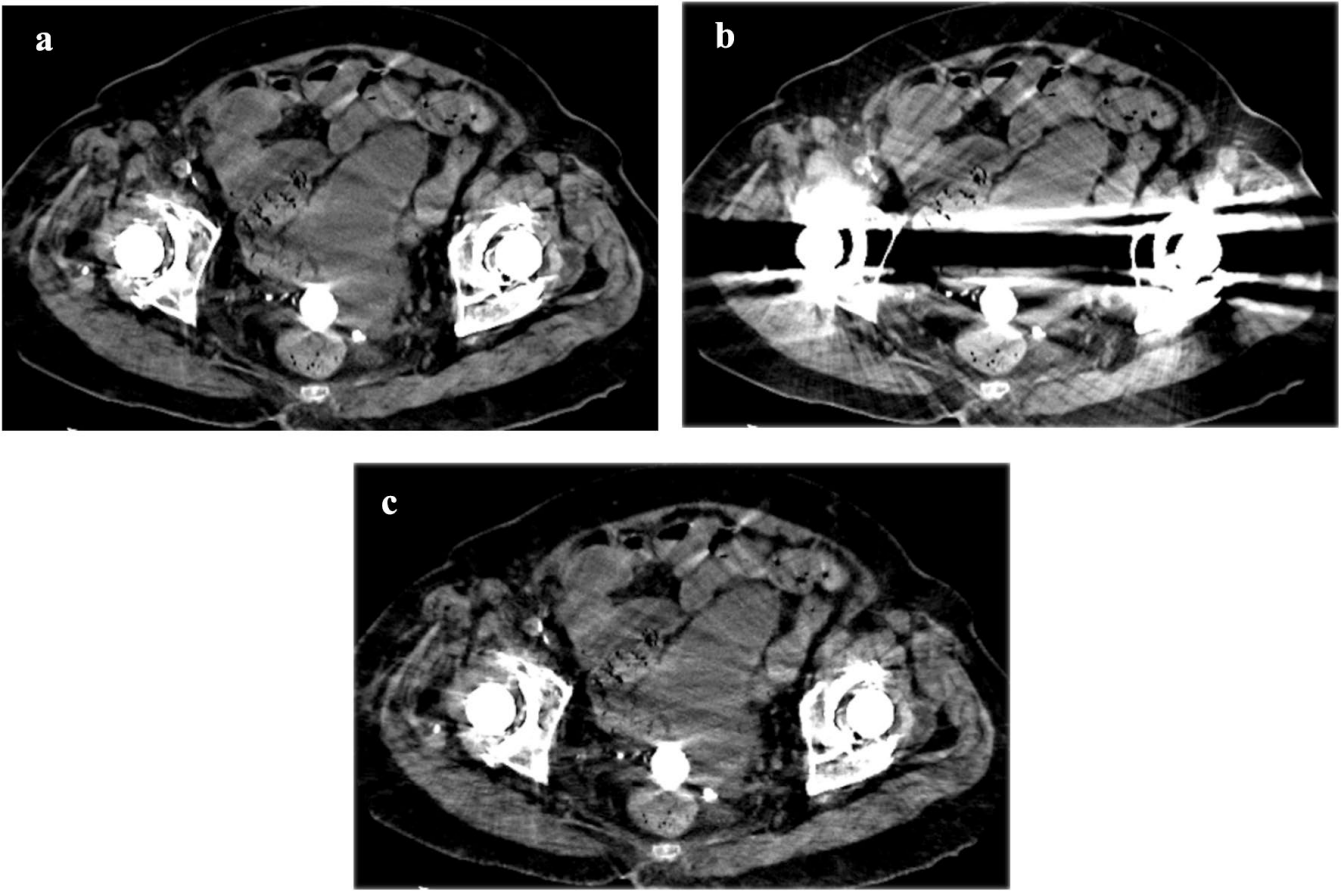
Axial unenhanced CT images reconstructed using DLR-S (**a**), DLR (**b**), and IR-S (**c**) of an 84-year-old woman. The degree of metal artifacts (DLR-S/DLR/IR-S) was rated as 4 (minimal artifacts)/1 (severe artifacts in a large area)/3 (moderate artifacts) by reader 1. It was rated 3 (moderate artifacts)/1 (severe artifacts in a large area)/3 (moderate artifacts) by reader 2. Bladder delineation (DLR-S/DLR/IR-S) was rated 5 (clear)/1 (unrecognizable)/2 (noticeably blurred) by reader 1. Reader 2 rated it as 4 (slightly blurred)/3 (moderately blurred)/4 (slightly blurred). A calcified structure was observed in all the reconstruction algorithms (**a**–**c**). However, the structure location was recognizable only on the DLR-S (**a**) and IR-S (**c**) images

**Fig. 4:**
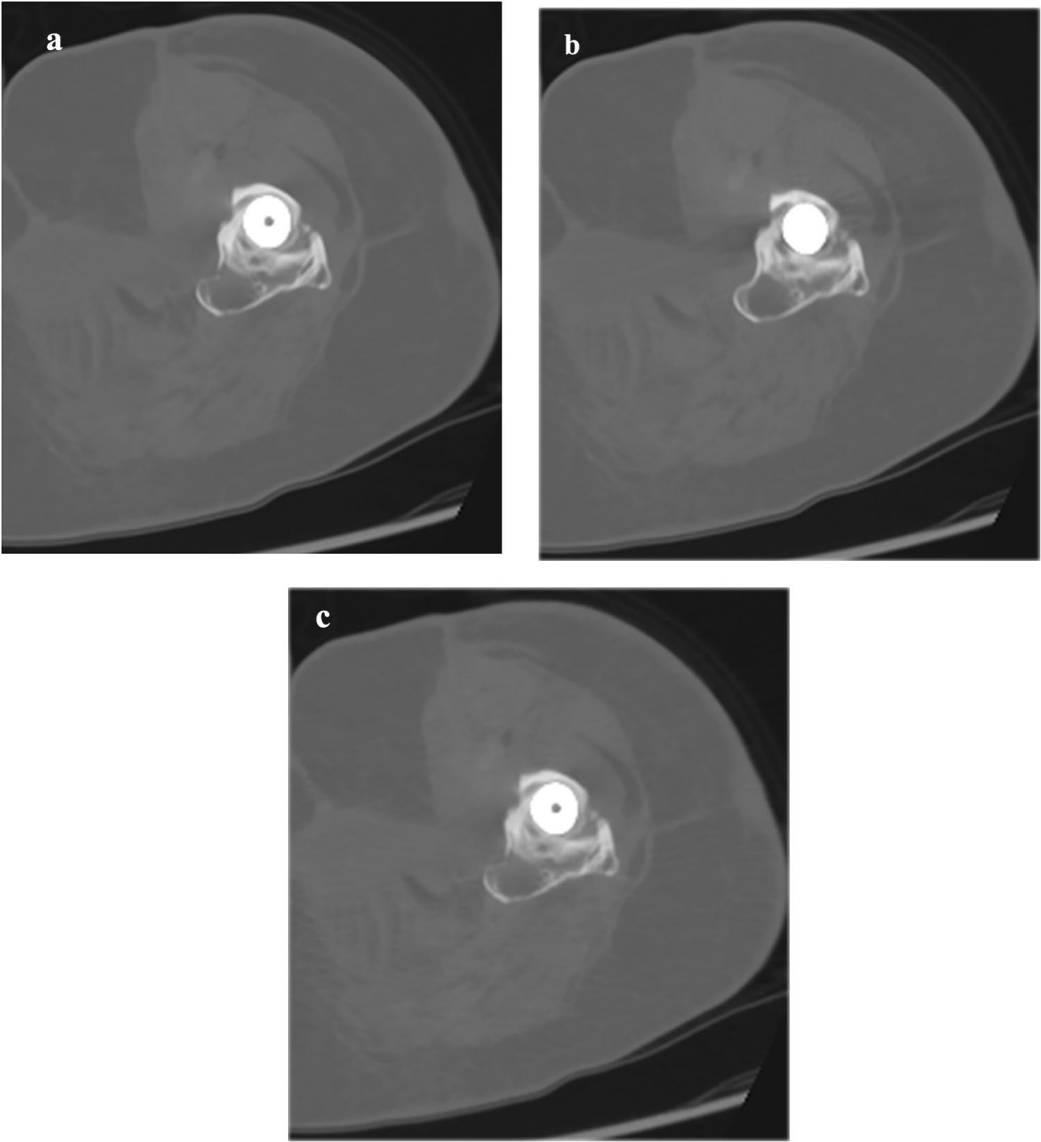
Axial unenhanced CT images reconstructed using DLR-S (**a**), DLR (**b**), and IR-S (**c**) of a 72-year-old woman. Bone delineation (DLR-S/DLR/IR-S) was rated 5 (clear)/4 (slightly blurred)/4 (slightly blurred) by reader 1. Reader 2 rated it as 4 (slightly blurred)/3 (moderately blurred)/3 (moderately blurred)

Detailed results of the one-by-one qualitative image analysis are summarized in Table 2. The degree of metal artifacts in the DLR-S images was significantly reduced compared with that in DLR and IR-S (*p* < 0.010), except for DLR-S *vs.* IR-S in reader 2’s evaluation (*p* = 1.000) (Figs. 2 and 3). The image noise of DLR-S was rated as significantly reduced compared with that of IR-S by both readers (*p* < 0.001). However, there was no significant difference in subjective image noise between DLR-S and DLR (*p* > 0.111). The depiction of all the structures in DLR-S was rated as significantly improved compared to with that in DLR by both readers (*p* < 0.014). Compared with the IR-S images, the DLR-S images were significantly better in depicting pelvic structures and nearby bones in all cases as rated by reader 1 (*p* < 0.029), but not significantly different as rated by reader 2 (*p* > 0.114). The interobserver agreements between both readers were good or excellent (0.614–0.813) for structural depiction excluding bone (Fig. 4), moderate (0.475) for depiction of bone, good (0.786) for artifacts, and moderate (0.472) for noise. The intraobserver agreements were good or excellent for structural depiction (0.615–0.896) excluding bone (moderate, 0.514–0.582), good to excellent for artifacts (0.658–0.831), and moderate to good for noise (0.423–0.679).

**Table 2:** Results for one-by-one qualitative image analyses

	Number of patients for each score (1/2/3/4/5) [total score]			Comparisons (*p*-values)		Intraobserver agreement	Interobserver agreement
	DLR-S	DLR	IR-S	DLR-S *vs.* DLR	DLR-S *vs.* IR-S		
Depiction of structures							
Bladder							
Reader 1	0/2/11/8/5 [94]	6/13/2/0/5 [63]	0/4/17/3/2 [81]	< 0.001*	0.027*	0.878	0.755
Reader 2	0/2/10/12/2 [92]	2/11/6/3/4 [74]	0/4/10/12/0 [86]	0.005*	0.288	0.719	
Ureter							
Reader 1	0/2/8/10/6 [98]	15/4/1/1/5 [55]	1/3/15/5/2 [82]	< 0.001*	0.003*	0.878	0.614
Reader 2	1/2/13/6/4 [88]	3/8/7/6/2 [74]	0/3/12/9/2 [88]	0.010*	1.000	0.615	
Rectum							
Reader 1	0/0/9/9/8 [103]	1/9/8/3/5 [80]	0/3/9/12/2 [91]	< 0.001*	0.010*	0.896	0.642
Reader 2	0/0/3/17/6 [107]	0/2/11/8/5 [94]	0/2/4/15/5 [101]	0.004*	0.191	0.715	
Rectovesical/rectouterine pouch							
Reader 1	0/1/8/9/8 [102]	2/8/7/4/5 [80]	0/3/9/12/2 [91]	< 0.001*	0.017*	0.877	0.707
Reader 2	1/2/7/15/1 [91]	5/5/8/6/2 [73]	1/3/9/11/2 [88]	0.014*	1.000	0.723	
Pelvic sidewall							
Reader 1	0/2/11/7/6 [95]	16/5/0/2/3 [49]	0/7/10/7/2 [82]	< 0.001*	0.010*	0.889	0.813
Reader 2	1/3/11/9/2 [86]	14/5/1/2/4 [55]	1/4/14/4/3 [82]	< 0.001*	0.534	0.869	
Bone							
Reader 1	0/0/4/13/9 [109]	0/2/15/5/4 [89]	0/0/7/16/3 [100]	< 0.001*	0.029*	0.514	0.475
Reader 2	0/0/13/12/1 [92]	0/4/16/6/0 [80]	0/0/19/7/0 [85]	0.006*	0.114	0.582	
Metal artifact and image noise							
Metal artifact							
Reader 1	0/1/11/13/1 [92]	9/11/3/3/0 [52]	0/2/20/4/0 [80]	< 0.001*	0.010*	0.831	0.786
Reader 2	0/1/21/4/0 [81]	7/16/0/3/0 [51]	0/1/22/3/0 [80]	< 0.001*	1.000	0.658	
Image noise							
Reader 1	0/0/7/13/6 [103]	1/2/10/8/5 [92]	0/7/16/3/0 [74]	0.111	< 0.001*	0.423	0.472
Reader 2	0/1/10/14/1 [93]	0/2/9/15/0 [91]	0/18/8/0/0 [60]	1.000	< 0.001*	0.679	

*DLR* deep learning reconstruction, *DLR-S* deep learning reconstruction with single-energy metal artifact reduction, *IR-S* hybrid iterative reconstruction with single-energy metal artifact reduction, The Wilcoxon signed-rank test was used to perform comparisons

^*^Statistically significant difference (*p* < 0.05)

### Qualitative image analyses (side-by-side)

Details of the side-by-side qualitative image analyses are summarized in Table 3. The results indicated that DLR-S was significantly better than IR-S in terms of the artifact and overall image quality.

**Table 3:** Results for side-by-side qualitative image analyses

	Mean (95% confidence interval) for DLR-S *vs.* IR-S score	
	Reader 1	Reader 2
Metal artifact	1.269 (1.025–1.513)	1.346 (1.092–1.600)
Overall image quality	1.808 (1.645–1.970)	1.846 (1.698–1.995)

*DLR-S* deep learning reconstruction with single-energy metal artifact reduction, *IR-S* hybrid iterative reconstruction with single-energy metal artifact reduction, Lower limit of 95% confidence interval is beyond 0 for all, which indicates that the DLR-S was rated as significantly better than IR-S in terms of metal artifact and overall image quality by both the readers

## Discussion

In this study, the use of SEMAR successfully reduced metal artifacts in DLR images, which resulted in better depiction of pelvic structures in CT images for patients with metal hip prosthesis. When compared with those in IR-S, metal artifacts and image noise were also reduced in DLR-S, and this was associated with significantly better overall image quality in DLR-S than in IR-S.

SEMAR is an algorithm known to reduce metal artifacts in CT images for patients with metals [4–7]. This algorithm can be applied to CT source data retrospectively (i.e., SEMAR can be used after the CT examination completion). In previous reports, SEMAR has been applied to hybrid IR CT images. For hybrid IR images, the use of SEMAR allowed significant metal artifact reduction and resulted in better depiction of pelvic structures in patients with hip prostheses [6, 7]. In the current study, we applied SEMAR to DLR images. In line with previous reports, the artifact was also successfully reduced in DLR-S images compared with that in DLR. We also compared DLR-S with IR-S images. In quantitative and side-by-side qualitative analyses, the metal artifact was found to be significantly reduced in DLR-S compared with that in IR-S. However, in one-by-one qualitative analyses, one reader found no significant difference in metal artifact between DLR-S and IR-S, while the other reader rated DLR-S was significantly better than IR-S. This indicated that DLR-S is surely better in reducing metal artifacts than IR-S, although the difference might be perceived as relatively small for some radiologists.

The DLR is a relatively new reconstruction algorithm that is known to improve the image quality of CT compared with hybrid IR algorithms [11]. This reconstruction algorithm is expected to be widely used in daily clinical practice. As described above, DLR can be used in combination with SEMAR. The comparison between DLR-S and IR-S confirmed that DLR-S provided significantly better noise reduction in both quantitative and qualitative assessments. This result would be compatible with a previous systematic review [11], which reported that DLR images showed a significantly higher signal-to-noise ratio than did hybrid IR images.

The depiction of all the evaluated pelvic structures in DLR-S was rated as significantly better than that in DLR by both readers, which would be due to the reduced metal artifacts in DLR-S. As for the comparison between DLR-S and IR-S, the two readers were divided in their evaluation regarding the depiction of pelvic structures and nearby bones. The differences in diagnostic imaging experience might be one of the reasons for this. The other possible reason would come from the fact that the structure depiction is affected by several factors including noise and artifact. The emphasized factors in judging the structure depiction might have differed from reader to reader. However, the side-by-side evaluation of the images showed an improvement in overall image quality between the DLR-S and IR-S images for both readers. This suggests that DLR-S would be a more suitable solution to be used in daily clinical practice than IR-S for CT examination in patients with metal hip prostheses.

Our study has some limitations. First, the results of this study cannot be directly applied to CT images obtained with scanners from other vendors since various products have been developed by various vendors for DLR technology and SEMAR is not necessarily available for CT scanners other than those from Canon Medical Systems. Second, owing to the retrospective nature of this study, we did not take into account the material of the metal prosthesis. Third, because the number of patients included in this study was relatively small, no subgroup analysis was performed between patients with unilateral and bilateral prostheses. Fourth, to reduce the number of comparisons in the same evaluation terms, we did not perform side-by-side qualitative analyses for the structure depiction between DLR-S and IR-S. However, we evaluated the overall image quality between them. Finally, because it was relatively hard to find the patients with specific diseases who also have metal hip prostheses, lesion detection performance was not evaluated. However, because the DLR-S artifact was found to be significantly improved compared with the DLR and IR-S artifacts from the current study, future studies focusing on lesion detection performance would be warranted.

In conclusion, our results suggested that the DLR method with SEMAR has the potential to significantly reduce metal artifacts in pelvic CT for patients with metal hip prostheses compared with conventional IR methods with SEMAR, allowing significantly better overall image quality. In addition, DLR with SEMAR reduced metal artifacts compared with DLR, which resulted in better depiction of pelvic structures. Therefore, DLR-S would be a better reconstruction algorithm in pelvic CT images for patients with metal hip prostheses in daily clinical practice than DLR or IR-S.
